# Plasmablastic lymphoma of the oral cavity with breast recurrence: a case report

**DOI:** 10.1186/s13104-015-1132-x

**Published:** 2015-05-02

**Authors:** Zarka Samoon, Romana Idrees, Nehal Masood, Tayyaba Zehra Ansari

**Affiliations:** Department of Oncology, The Aga Khan University Hospital, Stadium Road, PO BOX: 3500, Karachi, 74800 Pakistan; Department of Pathology, The Aga Khan University Hospital, Stadium Road, PO BOX: 3500, Karachi, 74800 Pakistan

**Keywords:** Plasmablastic lymphoma, Breast, Treatment

## Abstract

**Background:**

Plasmablastic lymphoma is an aggressive variant of diffuse large B cell lymphoma, mostly found in the oral cavity and associated with human immunodeficiency virus. There are no clear guidelines for its treatment. Therapies more intensive than cyclophosphamide, doxorubicin, vincristine, and prednisone are not associated with a prolonged survival. Lymphomas of the breast are rare, in one series representing 0.14% of all female breast malignancies, with diffuse large B cell lymphoma comprising up to 55% of all cases. Only one case of plasmablastic lymphoma involving the breast has been reported in the literature.

**Case presentation:**

A 30 year old Pakistani woman, presented with a small nodule in the floor of the mouth. An excisional biopsy revealed CD20, CD3, and CD117 negative and CD138, CD79a, CD56, MUM1/IFR4 and CD30 positive lesion with Ki-67 of 60% with cells which were plasmablastic in appearance. The morphological and immunohistochemistry features were consistent with plasmablastic lymphoma. The staging scans did not reveal any lymphadenopathy and the bone marrow biopsy and human immunodeficiency virus test were both negative. After treatment with four courses of CHOP and later radiation to the floor of the mouth, her disease was in complete remission. Two months later, she presented with velvety red lesions in both breasts and its trucut biopsy was consistent with plasmablastic lymphoma. Her CT scans revealed multiple nodules involving both breasts with no lymphadenopathy. The bone marrow was now positive for disease. Her disease continued to progress despite second and third line chemotherapy with DHAP (dexamethasone, cisplatin and cytarabine) and ICE (ifosfamide, carboplatin and etoposide) respectively. Her last CT scans revealed progressive disease with new lung lesions. The patient decided to opt for best supportive care.

**Conclusion:**

To our knowledge this is the second report of plasmablastic lymphoma involving the breast. The patient who was human immunodeficiency virus negative and immune competent had progressive disease despite three lines of chemotherapies with an overall survival (to date) of 15 months.

## Background

Plasmablastic lymphoma (PBL) is a rare variant of Diffuse large B cell lymphoma, mostly found in the oral cavity [1] and associated with human immunodeficiency virus (HIV). Other rare sites of its occurrence include oropharynx, nasopharynx, stomach, skin, lungs, sacrococcygeal, pericardial, anorectal, nasal, paranasal or submandibular regions [2]. Lymphoma of the breast is a rare entity; representing 0.14% of all female breast malignancies [3]. However, amongst the breast lymphomas, the most common type is diffuse large B cell lymphoma involving up to 55% of all cases. This subtype usually occurs in women of older age, with slight predominance of right side [3]. Other rare types of lymphomas to involve the breast are Burkitt’s lymphoma, Extranodal marginal zone B cell lymphoma of mucosa associated lymphoid tissue (MALT) type, follicular lymphoma, and very rarely, T-cell lymphomas. Lymphomas involving the breast are very rare, yet very aggressive. Hence they need to be treated with combination chemotherapy with or without local irradiation. In case of progression or relapse, the response to salvage therapy is poor [3].

Herein, we present a case of a 30 year old HIV-negative immunocompetent woman, who presented with a nodule in the floor of the mouth. A histopathological diagnosis of PBL was made and she was treated with chemotherapy followed by local radiation. She later presented with disease relapse in the breast. To our knowledge this is the second report of PBL involving the breast [4].

## Case presentation

**Figure 1:**
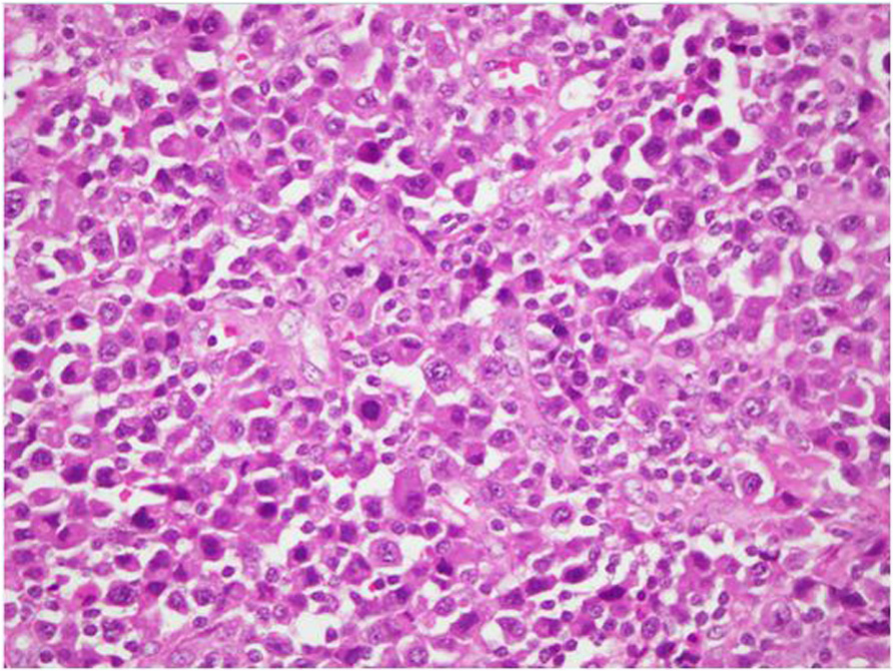
H&E (hematoxylin and eosin) image shows large sheets of mostly large plasmacytoid appearing mononuclear cells.

**Figure 2:**
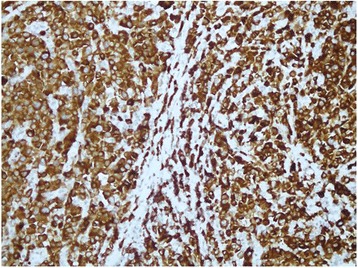
CD79a, immunohistochemical stain demonstrates plasmacytic differentiation.

**Figure 3:**
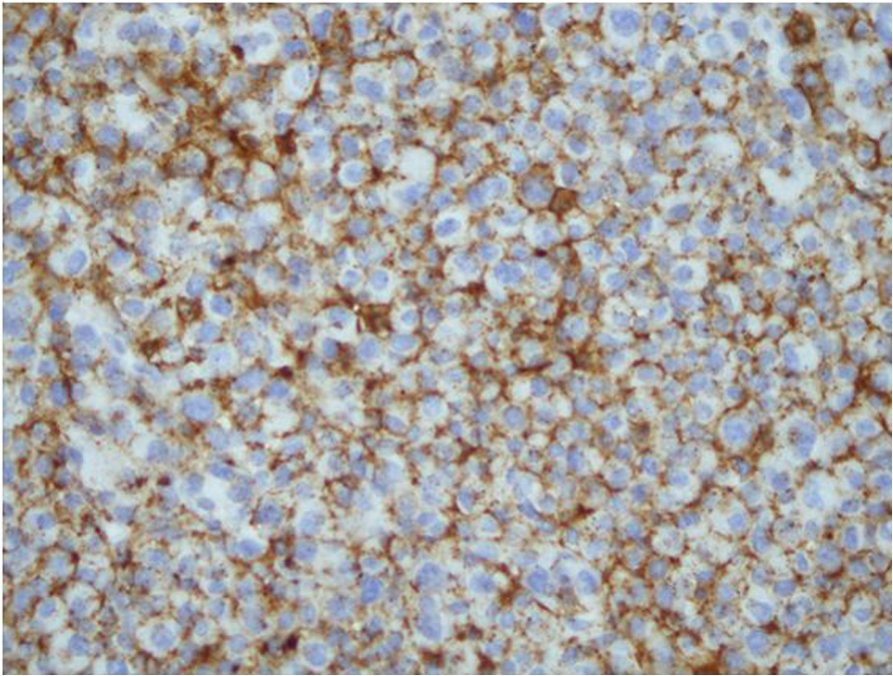
CD138, immunohistochemical stain demonstrates plasmacytic differentiation.

**Figure 4:**
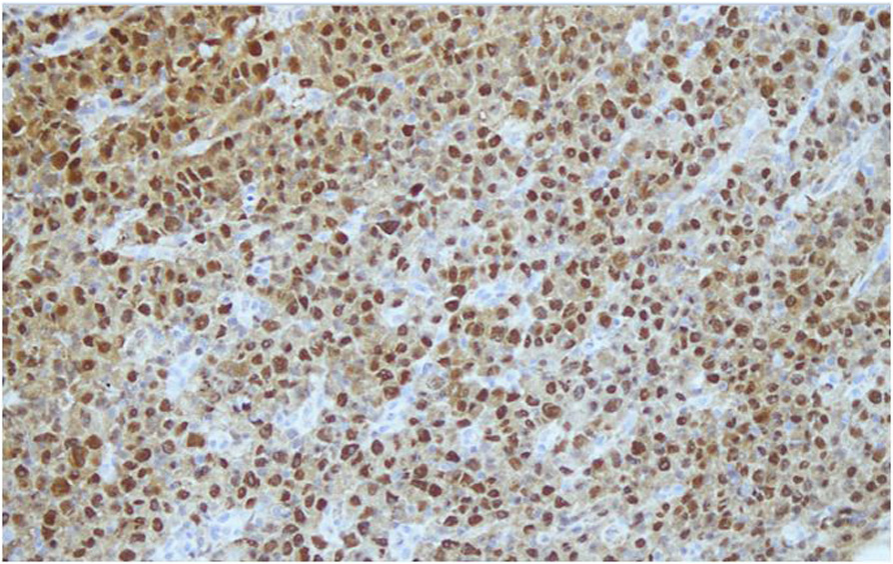
MUM1/IRF4 shows positivity in neoplastic cells.

**Figure 5:**
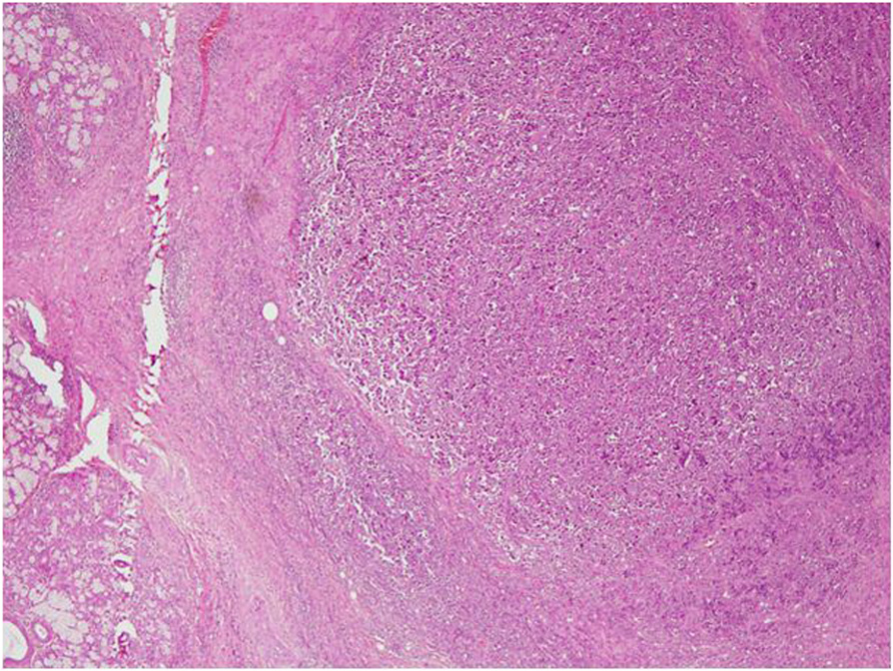
Breast biopsy shows involvement by plasmablastic lymphoma.

A 30 year old Pakistani woman, presented with few weeks history of dysphagia. Clinically she was well preserved and examination revealed small firm 1 cm nodule on the floor of the mouth. The excisional biopsy thus performed revealed neoplastic cells, which were arranged in diffuse sheets and were plasmablastic in appearance with vesicular nuclei, and centrally located prominent nucleolus, with abundant basophilic cytoplasm (Figure 1). On immunohistochemistry these plasmacytoid cells were negative for conventional B cell marker CD20 while pan T CD 3 was also negative. These cells showed strong positivity for CD138, CD79a, CD56, and MUM1/IFR4 with Ki-67 of approximately 60% (Figures 2, 3 and 4). The morphological and immunohistochemistry features were consistent with PBL. The rest of her systemic examination was unremarkable. The CT scan of head, neck, chest, abdomen and pelvis with contrast did not reveal any lymphadenopathy and the bone marrow biopsy was negative for disease. Patient was tested to be negative for HIV. She did not have any other condition which could compromise her immunity. Her last pregnancy was about eight years back. She was treated with standard CHOP (cyclophosphamide, doxorubicin, vincristine and prednisolone) chemotherapy. She had no evidence of disease at the time of initiation of chemotherapy and remained in remission after 4 courses of CHOP; hence, treatment was consolidated with local radiation. Two months later, she presented with self-discovered lumps in both breasts. It was not associated with pain or nipple discharge. Examination revealed diffuse erythema of the skin of both breasts with peau-d-orange and velvety red lesions. The rest of her systemic examination was unremarkable. She did not have any high risk features for breast cancer such as history of oral contraceptive usage, personal or family history of breast cancer. A trucut biopsy of the breast lesions was consistent with the prior diagnosis of plasmablastic lymphoma (Figure 5). Her CT scans of head, neck, chest, abdomen and pelvis with contrast revealed multiple rounded soft tissue density nodules involving both breasts with no lymphadenopathy. The bone marrow was now positive for disease and was weakly positive for CD56. She received four courses of DHAP (dexamethasone, cisplatin and cytarabine). She had good clinical and radiological response to chemotherapy initially however by the end of four courses, her breast lesions recurred. She was then switched to third line chemotherapy with ICE (ifosfamide, cyclophosphamide and etoposide). Her breast lesions initially regressed but grew before next cycle of chemotherapy in 2 weeks’ time. Her CT scans of head, neck, chest, abdomen and pelvis with contrast revealed progressive disease with increase in the number of breast lesions with new development of pleural effusion and multiple lung nodules bilaterally. Further treatment options were discussed but considering futility of treatment only best supportive care was pursued and she went back to her native town.

## Discussion

Plasmablastic lymphoma is usually associated with HIV infection, where it accounts for 2.6% of all HIV related Non-Hodgkin’s lymphomas [5]. However it may be present in patients who are HIV negative but otherwise immunocompromised, such as patients who have undergone solid organ transplantation or bone marrow transplantation or those with autoimmune disease [2]. It was in 1997 when Delecluse and colleagues reported the first series of sixteen patients with diffuse large B cell lymphoma of oral cavity with unique immunohistochemical features [6]. Based on their morphologic and immunohistologic features these tumors were named plasmablastic lymphoma [6]. PBL is said to be derived from post germinal-center activated B cells, which are terminally differentiated, believed to be in evolution from immunoblast to plasma cell [7]. PBL reveal little to no expression of leukocyte common antigen (CD45) or the B-cell markers CD20 and CD79a. However, there is almost unanimous expression of plasma cell markers VS38c, CD38, multiple myeloma oncogene-1 (MUM1/IRF4), and CD138 (syndecan-1) [8].

Plasmablastic lymphoma has a very aggressive clinical course with a median overall survival of about fifteen months [8]. There are no clear guidelines for its treatment. Therapies more intensive than CHOP (cyclophosphamide, doxorubicin, vincristine, and prednisone) are not associated with a prolonged survival [9]. There are several features associated with better survival, which include the clinical stage, use of antiretroviral therapy (in HIV patients) and achievement of complete response to chemotherapy [9]. Autologous stem cell transplantation has been tried in such patients however the follow up data has been too short to conclude it being effective [10]. Bortezomib, a proteasome inhibitor may be a new therapeutic option for plasmablastic lymphoma, which has resulted in dramatic responses, however, the response has not been sustained [11].

## Conclusion

In conclusion we report a case of plasmablastic lymphoma of the breast which initially presented with a hard palate lesion. Our case was unique in presentation that there was no evidence of disease after excisional biopsy of hard palate and at recurrence breast was the only site. Later however she also developed bilateral lung nodules and pleural effusion. To our knowledge this is the second reported case of PBL involving the breast [4] and the first case report of how PBL of the breast was treated.

## Consent

Written informed consent was obtained from the patient for publication of this case report and any accompanying images. A copy of the written consent is available for review by the Editor-in-Chief of this journal.

## Abbreviations

CHOP: Cyclophosphamide, Doxorubicin, Vincristine and Prednisolone
DHAP: Dexamethasone, cisplatin and cytarabine
HIV: Human immunodeficiency virus
ICE: Ifosfamide, carboplatin and etoposide
PBL: Plasmablastic lymphoma
